# A Highly Selective and Strong Anti-Interference Host-Guest Complex as Fluorescent Probe for Detection of Amantadine by Indicator Displacement Assay

**DOI:** 10.3390/molecules23040947

**Published:** 2018-04-18

**Authors:** Linzhao Zhu, Zhiyong Zhao, Xiongzhi Zhang, Haijun Zhang, Feng Liang, Simin Liu

**Affiliations:** The State Key Laboratory of Refractories and Metallurgy, School of Chemistry and Chemical Engineering, Wuhan University of Science and Technology, Wuhan 430081, China; zlz92400@163.com (L.Z.); zhaozhiyong@wust.edu.cn (Z.Z.); zxz15872406033@163.com (X.Z.); zhanghaijun@wust.edu.cn (H.Z.); feng_liang@wust.edu.cn (F.L.)

**Keywords:** fluorescent probe, amantadine, cucurbit[7]uril, host-guest interaction, indicator displacement assay

## Abstract

Amantadine (AMA) and its derivatives are illicit veterinary drugs that are hard to detect at very low concentrations. Developing a fast, simple and highly sensitive method for the detection of AMA is highly in demand. Here, we designed an anthracyclic compound (ABAM) that binds to a cucurbit[7]uril (CB[7]) host with a high association constant of up to 8.7 × 10^8^ M^−1^. The host-guest complex was then used as a fluorescent probe for the detection of AMA. Competition by AMA for occupying the cavity of CB[7] allows ABAM to release from the CB[7]-ABAM complex, causing significant fluorescence quenching of ABAM (indicator displacement assay, IDA). The linear range of the method is from 0.000188 to 0.375 μg/mL, and the detection limit can be as low as 6.5 × 10^−5^ μg/mL (0.35 nM). Most importantly, due to the high binding affinity between CB[7] and ABAM, this fluorescence host-guest system shows great anti-interference capacity. Thus, we are able to accurately determine the concentration of AMA in various samples, including pharmaceutical formulations.

## 1. Introduction

**Figure 1:**
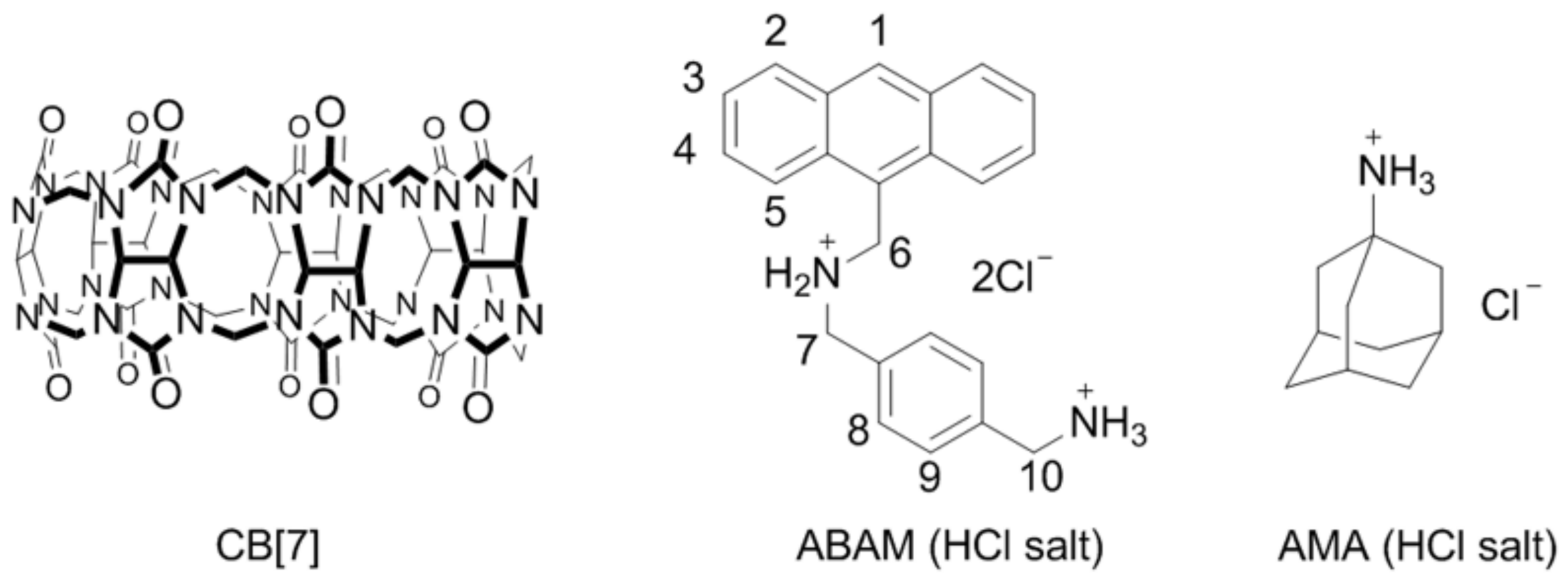
The structures of CB[7], ABAM, and AMA.

Amantadine (AMA, Figure 1) was first found by Davies in 1964, and was used to treat influenza virus [1]. Because of its effective treatment and low cost, AMA was recognized as the first anti-influenza drug by the U.S. Food and Drug Administration (FDA) in 1966 [2,3]. With its advantages, AMA has been mixed with fodder, aiming to prevent and treat animal diseases, especially in chicken farming [4]. This kind of drug abuse results in the accumulation of AMA in the human body, causing various kinds of poisonous effects. Additionally, the abuse of AMA may also cause virus resistance and virus variation, which have bad effects on human treatment [5]. Because of these potential risks, AMA has been banned in livestock farming by USA and China since 2005 [6,7]. So far, lots of assays have been reported for the detection of AMA, including near-infrared spectroscopy [8,9], HPLC-MS/GC-MS [6,10,11,12,13,14,15,16], micellar electrokinetic chromatography (MEKC) [17], capillary electrophoresis [18,19], and potentiometry [20,21]. However, those methods normally require complicated, expensive equipment and labor-intensive sample preparation procedures. Moreover, some of them are not sensitive enough. Spectrofluorimetry is regarded as one of the most convenient analytical techniques in pharmaceutical analysis, owing to its inherent simplicity, high sensitivity, and availability in most quality-controlled and clinical laboratories. Considering that the aqueous solution of AMA has no native fluorescence, the concentration of AMA cannot be directly determined through the normal fluorimetric method. Therefore, spectrofluorometric methods have been developed via the derivatization of AMA with a chromophore [22,23,24].

Cucurbit[*n*]uril (CB[*n*], *n* = 5–8, 10, 13–15) is a family of macrocyclic compounds with a hydrophobic cavity and 2*n* carbonyl group on the portals [25,26,27]. As a novel synthetic host molecule, CB[*n*] shows its molecular recognition properties towards cationic and neutral guests with high selectivity and high affinity, and has been widely utilized in applications in nanoreactors/catalyst, supramolecular materials, and drug delivery/biomedicine [28,29,30,31,32,33,34,35,36]. CB[7] (Figure 1), one of the most studied members of the CB[*n*] family, shows its abilities to recognize a variety of guests with high association constant in aqueous solution, such as adamantylamine derivatives [37,38]. Due to the ability to alter the photophysical properties of fluorescent dyes [39], CB[7] has been applied to form a host-dye complex, which has been further used for determining analyte concentrations via indicator displacement assays (IDA) [40].

**Scheme 1:**
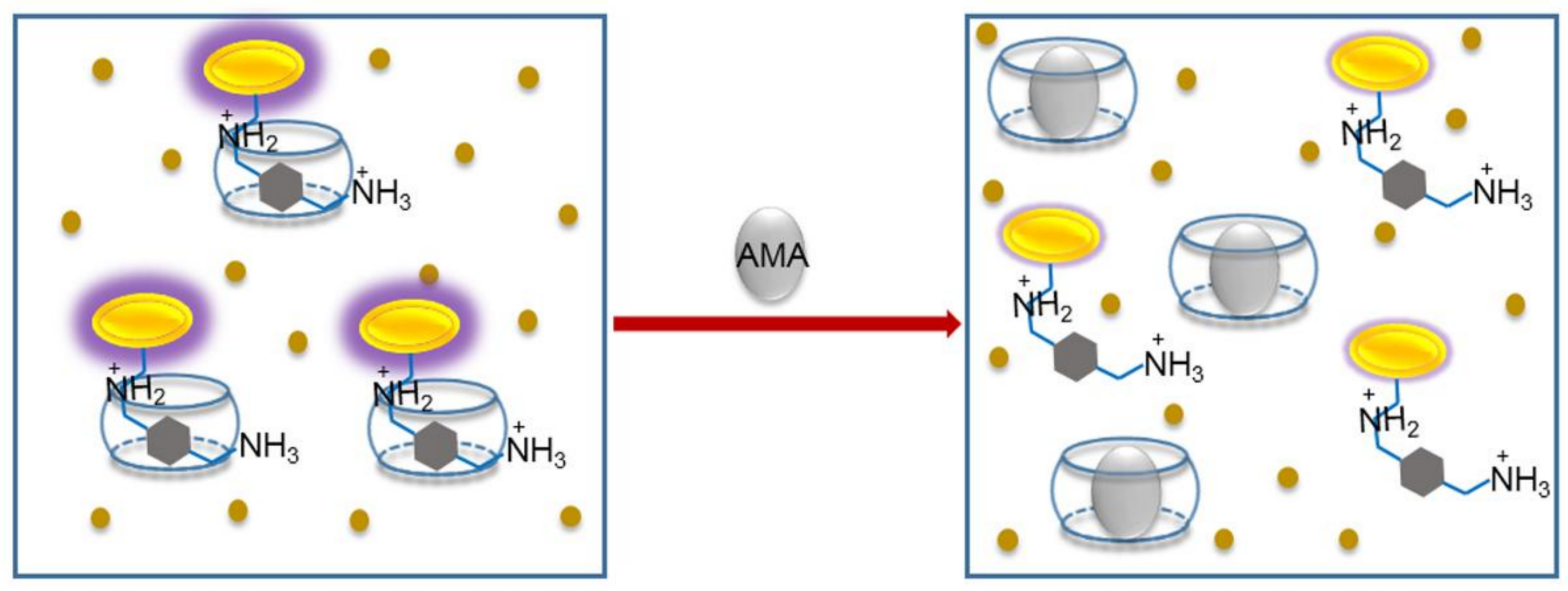
Schematic representation of the principle of indicator displacement assay for the detection of AMA using the complex CB[7]·ABAM as a fluorescent probe.

The first host-guest fluorescent probe system aiming at detecting AMA was reported in 2012 [41]. A natural isoquinoline alkaloid-coptisine (COP) was selected as the indicator, and the system was quite sensitive to AMA. Owing to the week binding interaction between COP and CB[7] (*K*_a_ = 1.86 × 10^4^ M^−1^), other non-target drug molecules may also displace COP from the complex and significantly skew measurement results. In another example, a bis-pyridine fluorescent molecule is used as the indicator [42]. But still, the binding interaction between the indicator and CB[7] is not strong enough (*K*_a_ = 9.44 × 10^4^ M^−1^). In order to achieve the detection of AMA with high selectivity, sensitivity, and anti-interference capacity, we designed a fluorescence indicator *N*-(4-(aminomethyl)benzyl)-1-(anthracen-9-yl)methanamine (ABAM, Figure 1). We expect that aminomethyl-benzyl moiety could be encapsulated in CB[7] [37]; anthracene moiety could stay out of the cavity of CB[7] and its fluorescence signal could change upon complexation/decomplexation of ABAM with CB[7]. Since the binding constants of CB[7]·xylylenediamine and CB[7]·AMA are 1.84 × 10^9^ M^−1^ and 4.2 × 10^12^ M^−1^, respectively [37], we expect the system could show high anti-interference capacity and maintain high sensitivity (Scheme 1). After making the new indicator, we investigated the binding behaviour of ABAM with CB[7], including the emission properties of ABAM upon encapsulation in CB[7], and plotted the standard curves upon the fluorescence change of CB[7]-ABAM system with addition of AMA. Finally, we determined the concentration of AMA in different samples, including mixture samples even pharmaceutical formulations.

## 2. Results and Discussion

### 2.1. Binding Studies of the Fluorescent Indicator with CB[7]

**Scheme 2:**
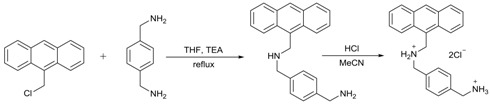
Synthesis of fluorescence indicator ABAM (as HCl salt).

**Figure 2:**
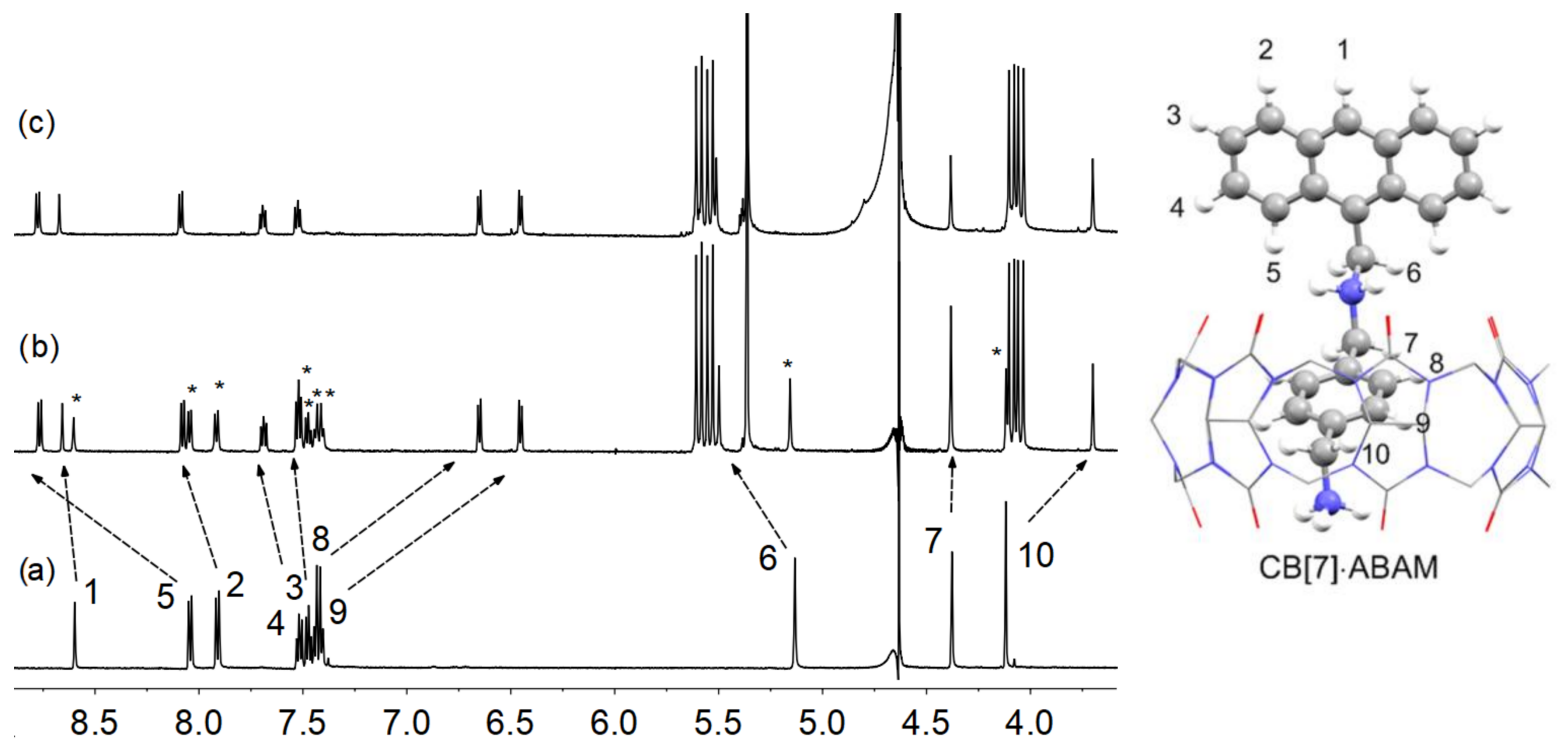
^1^H NMR spectra (600 MHz, D_2_O, 298 K) of: (**a**) free ABAM (1.0 mM); (**b**) 2:1 mixture of ABAM and CB[7] (resonances of free ABAM are marked with *); (**c**) 1:1 mixture of ABAM and CB[7]. The plausible schematic configuration of the inclusion complex CB[7]·ABAM.

The fluorescence indicator ABAM (as HCl salt) was synthesized by the reaction of 9-(chloromethyl) anthracene with 1,4-di(aminomethyl) benzene (Scheme 2 in Section 3). The binding between ABAM (as HCl salt) and CB[7] was first investigated by ^1^H NMR spectroscopy in D_2_O (Figure 2). As shown in Figure 2b, when the molar ratio between ABAM and CB[7] was 2:1, both free and bound peaks of ABAM molecule were observed, indicating the complexation-decomplexation processes between ABAM and CB[7] is slow exchange kinetics on the ^1^H NMR time scale. The resonance signals of H_8_, H_9_, as well as H_10_ protons shifted upfield significantly (Δδ = 0.79 ppm, 0.96 ppm, and 0.42 ppm respectively), suggesting the encapsulation of the 4-aminomethyl-benzyl moieties in the cavity of CB[7]. The signals of H_5_ and H_6_ protons shifted downfield significantly because of their location in the de-shielding region of CB[7]. The slight downfield shifts of H_1_-_4_ proton signals is caused by weakened π−π stacking interactions between anthracene moieties upon the encapsulation of ABAM in CB[7] [43]. Adding more than 1 eq. of CB[7] did not induce further chemical shifts of ABAM proton signals, indicating the 1:1 host-guest complexation between the indicator and CB[7]. Based on this, a plausible schematic configuration of the inclusion complex CB[7]·ABAM is given in Figure 2. Further, association constants of the CB[7]·ABAM complex as high as 8.7 × 10^8^ M^−1^ were obtained by ^1^H NMR competition experiment (Figure S4 and Table S1) [37].

**Figure 3:**
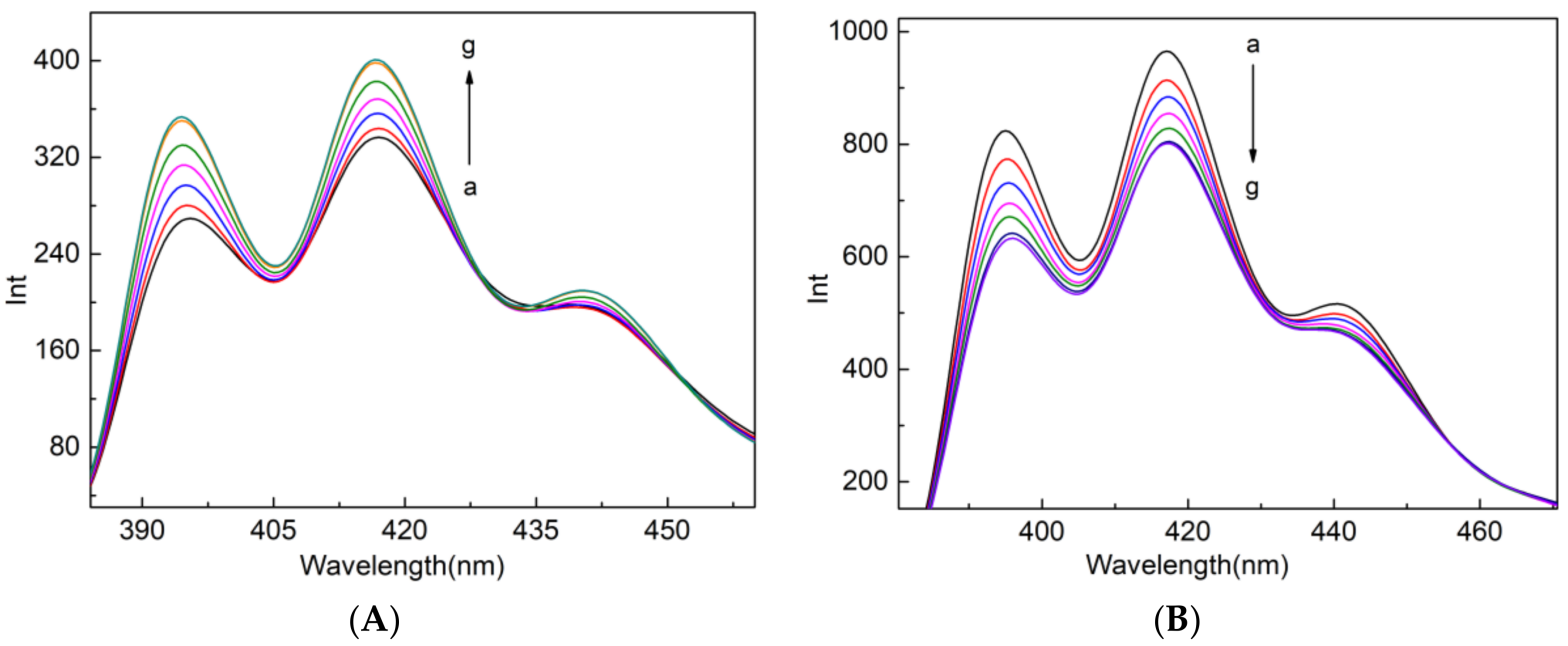
(**A**) Fluorescence spectra of the ABAM with addition of CB[7]: (a) 0; (b) 0.2 eq.; (c) 0.4 eq.; (d) 0.6 eq.; (e) 0.8 eq.; (f) 1.0 eq.; (g) 1.2 eq. ([ABAM] = 2 μM, pH = 4.70, λ_ex_/λ_em_ = 366/417 nm, the excitation and emission monochromator bandpasses were set at 15 nm and 3 nm, respectively). (**B**) Fluorescence spectra of the ABAM in the presence of CB[7] with addition of AMA: (a) 0; (b) 0.2 eq.; (c) 0.4 eq.; (d) 0.6 eq.; (e) 0.8 eq.; (f) 1.0 eq.; (g) 1.2 eq. ([CB[7]·ABAM] = 0.2 μM, pH = 4.70, λ_ex_/λ_em_ = 366/417 nm, the excitation and emission monochromator bandpasses were set at 10 nm and 6 nm, respectively).

Considering the pH value may have influence on fluorescence intensity changes in experiments, the pH value in all experiments for the detection of AMA was set as 4.70 using sodium acetate buffer (50 mM). The fluorescence changes of the indicator in the presence of CB[7] host were investigated. As shown in Figure 3A, with addition of CB[7] to the ABAM solution, the fluorescence intensity of ABAM was significantly increased. As with most introduced CB[7]-dye systems, the enhancement of fluorescence intensity of ABAM results from the dispersion of ABAM upon its inclusion in CB[7] [39]. Adding more than 1 eq. of CB[7] could not change fluorescence much, verifying the stable 1:1 host-guest complex formation of ABAM with CB[7]. These results are in agreement with the NMR consequences. Considering that protonation/deprotonation could influence the emission of dye molecules, we recorded the fluorescence of ABAM and ABAM·CB[7] under various pH values. The results indicate that the acidities of ABAM and CB[7]·ABAM are 8.8 and 11.5, respectively (Figure S5 and S6), which verifies that in the buffer (pH 4.70) the fluorescence changes of ABAM are completely irrelevant to the protonation/deprotonation of amino groups on ABAM. Additionally, the data proves the ability of CB[7] to increase the p*K*_a_ value of the encapsulated guest molecule [44].

### 2.2. Plots of Standard Curve

Since the binding constant of CB[7]·AMA is ~3.5 order of magnitude higher than that of CB[7]·ABAM, it is expected that AMA can easily displace ABAM from the complex CB[7]·ABAM. Indeed, adding AMA (as HCl salt) into the solution of CB[7]·ABAM complex caused an obvious decrease of fluorescence intensity of CB[7]-ABAM system (Figure 3B). Similarly, after adding more than 1 eq. of AMA, the fluorescence intensity no longer decreased, because all of the indicator had been squeezed out of the cavity of CB[7]. Determining the proper concentration of the host-guest fluorescence system is crucial. If the concentration of probe system is too low, the sensitivity and accuracy could be low. Conversely, the high concentration may not help in determining the optimum detection limit of the analyte. After several attempts, we found the widest AMA detection range when the concentration of the host-guest probe system was 0.2 μM.

**Figure 4:**
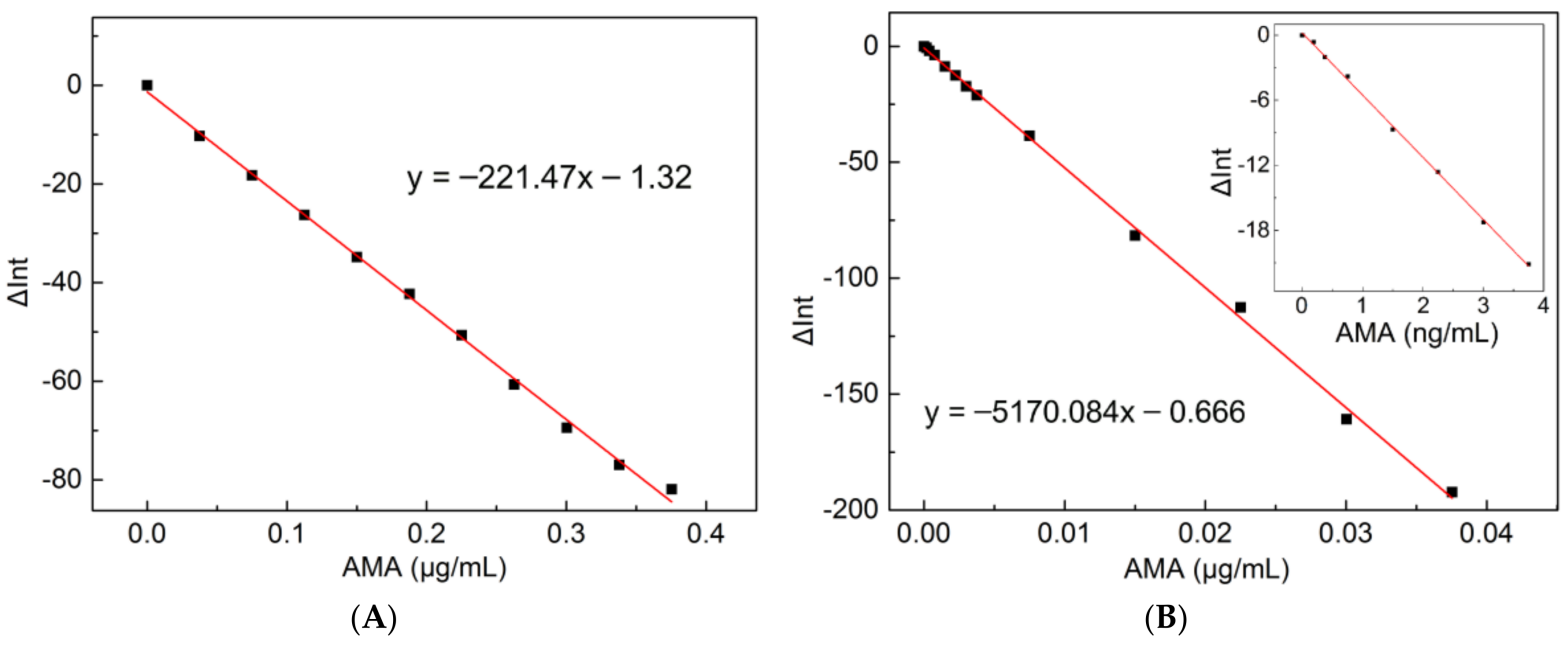
(**A**) The standard curve of AMA ranging from 0.0375 μg/mL–0.375 μg/mL ([CB[7]·ABAM] = 2.0 μM, pH = 4.70, λ_ex_/λ_em_ = 366/417 nm). (**B**) The standard curve of AMA ranging from 0.000188 μg/mL–0.0375 μg/mL ([CB[7]·ABAM] = 0.2 μM, pH = 4.70, λ_ex_/λ_em_ = 366/417 nm).

According to the changes of the fluorescence intensity of CB[7]-ABAM system as a function of the concentration of AMA, we plotted two standard curves for determining different ranges of AMA concentration (Figure 4) (the titration graphs are shown in Figures S7 and S8). When the concentrations of CB[7]-ABAM system are 2.0 μM (Figure 4A) and 0.2 μM (Figure 4B), the linear regression equations are *y* = −221.47*x* (μg/mL) − 1.32 and *y* = −5170.084*x* (μg/mL) − 0.666, respectively (*y* represents the change value of the fluorescence intensity, *x* represents the mass concentration of AMA). The correlation coefficients of two equations are 0.998 and 0.999, indicating good linearity. The linear ranges are 0.0375–0.375 μg/mL and 0.000188–0.0375 μg/mL for AMA, respectively. These equations can be directly used for the detection of AMA in real samples according to the change value of fluorescence intensity of CB[7]-ABAM system. With the detection limit as 6.5 × 10^−5^ μg/mL, this method proved to have higher sensitivity for detecting AMA than any other method reported in the literature (Table 1).

### 2.3. Detection of AMA in Different Samples

We firstly investigated the effect of interfering substances on this fluorescent probe system. Four different veterinary drugs—ribavirin, doxycycline, levofloxacin and florfenicol—were selected. From the fluorescence titration (Figure S9) we can see, after adding these interfering molecule mixtures to the host-guest probe system, that the fluorescence intensity didn’t show any obvious change. Then, we tried to analyse the simulative samples. The four veterinary drugs mentioned above were mixed into the solution of AMA to simulate a real sample. Three simulated samples were prepared in which the concentrations of AMA were 150 nM, 25 nM and 5 nM, respectively. The concentration of each interfering molecule was 100 times higher than AMA, which means the total concentration of interfering substances was 400 times. A highly concentrated solution of the host-guest probe system in 50 mM NaOAc buffer was prepared, and then diluted to 0.2 μM with the simulative sample. The fluorescence intensity values of the solution (FI_CB[7]-ABAM-AMA_) and the blank solution (FI_CB[7]-ABAM_) were measured at 417 nm with an excitation wavelength of 366 nm (Figure S10). Again, the change value of the fluorescence intensity (ΔFI) was put into the equation to calculate the concentration of AMA. In Table 2, the almost 100% recovery value indicates the system has strong anti-interference capacity, with high accuracy when the concentration of AMA in the sample was as low as 25 nM (4.69 ng/mL). Even when the concentration of AMA in the sample was as low as 5 nM (0.94 ng/mL), a recovery value of 95% is good enough at such a low concentration. These results indicate that the fitting equations are very practical.

Furthermore, the real pharmaceutical formulation was tested. The amantadine hydrochloride tablet was obtained from Northeast Pharm. Each tablet contains: acetaminophen 250 mg, AMA hydrochloride 100 mg, artificial bezoar 10 mg, caffeine 15 mg, and chlorpheniramine maleate 2 mg. The other accessories include starch, magnesium stearate, hydroxypropyl cellulose. Weighed tablets were carefully pulverized, placed and sonicated with water in a 100 mL calibrated flask, and then the solution was filtered. AMA hydrochloride was regarded as undergoing no loss in the filtering operation, due to its good water solubility in buffer solution (pH 4.70). The solution of AMA sample was further diluted to 100 nM, according to the AMA content per tablet in the drug instructions. The measuring operation was the same as that for the simulative samples. On the basis of this method, the detected concentration of AMA was 96.56 nM, and the recovery was up to 96.6% (Figure S11).

## 3. Materials and Methods

### 3.1. Materials

CB[7] was prepared according to the corresponding procedures in the literature [26]. Other compounds used in this study were purchased from commercial suppliers and used without further purification.

Synthesis of ABAM:

1,4-di(aminomethyl) benzene (0.48 g, 3.53 mmol) and triethylamine (0.45 mL, 3.11 mmol) were mixed in 30 mL dry THF in a flask, stirring at room temperature for an hour. After that, 9-(chloromethyl) anthracene (0.40 g, 1.76 mmol) was added slowly to the solution, and the mixture was refluxed overnight. The mixture was filtered and evaporated to yield a yellow solid, which was purified by column chromatography with a 20:1 mixture of methylene dichloride/methanol. The product (R_f_ = 0.34) was collected as a pale yellow solid-ABAM (340 mg, 60%). Dissolving the pure product into acetonitrile, bubbling hydrogen chloride gas in the solution, and then a white precipitate formed. The precipitate was collected and dried to give ABAM hydrochloride (390 mg, 94%). ^1^H NMR (600 MHz D_2_O) δ (ppm) = 4.12 (s, 2H, CH_2_), 4.38 (s, 2H, CH_2_), 5.14 (s, 2H, CH_2_), 7.42 (m, 2H, CH), 7.44 (d, 2H, CH), 7.43 (t, 2H, CH), 7.52 (t, 2H, CH), 7.92 (d, 2H, CH), 8.05 (d, 2H, CH), 8.60 (s, 1H, CH). ^13^C NMR (150 MHz D_2_O) δ (ppm) = 134.18, 130.94, 130.84, 130.27, 130.03, 129.67, 129.46, 129.18, 127.35, 125.27, 121.78, 119.78, 50.15, 42.59, 40.65. ESI-MS (positive ion): *m/z* 327.19 [ABAM + H^+^]^+^ (calcd. C_23_H_23_N_2_^+^ 327.18).

### 3.2. Instrumentation

NMR spectra (^1^H, ^13^C and COSY) were collected on Agilent 600 MHz DD2. Mass spectrometry was performed using a Bruker Agilent 1290-micrOTOF Q II. Fluorescence spectra were measured on a PerkinElmer LS-55 machine, with an excitation wavelength of 366 nm. 

### 3.3. Determination of K_rel_

We use Equation (1) to determine *K*_rel_ for the interaction of ABAM and *p*-xylylenediamine (PXDA) for CB[7]. For this purpose, we prepared a solution containing CB[7] (1.0 mM), ABAM (1.0 mM), PXDA (1.0 mM) and allowed it to reach equilibrium. Next, we determined the relative concentration of CB[7]·ABAM by integration of the appropriate resonances in the ^1^H NMR spectrum.

*K*_rel_ = ([CB[7]·PXDA][ABAM]_free_)/([CB[7]·ABAM][PXDA]_free_) (1)

[PXDA]_Total_ = 1mM = [PXDA]_free_ + [CB[7]·PXDA] (2)

[ABAM]_Total_ = 1mM = [ABAM]_free_ + [CB[7]·ABAM] (3)

[CB[7]·PXDA] = 1 − [CB[7]·ABAM](4)

## 4. Conclusions

In summary, we have designed a new indicator compound ABAM bearing benzylamine unit as the binding group for CB[7] host and anthracene group as the chromophore. The high association constant of ABAM with CB[7] of up to 8.7 × 10^8^ M^−1^ suggests the strong anti-interference performance of the CB[7]-indicator system; the strong emission of the fluorescent probe at low concentration implies the low detection limit of the system. Indeed, through indicator displacement assay, we were able to fit the data to get nice linear equations, which could then be used in real sample detection. Further investigations revealed that as we expected, this system shows a low detection limit (6.5 × 10^−5^ μg/mL, the lowest detection limit ever reported) and strong anti-interference capacity. In combination with its easy synthesis and purification process, this system could be of great significance to the detection of AMA concentration in drugs and other samples, such as animal meats.

## Figures and Tables

**Table 1:** Various methods and detection limits for the detection of AMA.

Technique	Sample	Linear Range (μg/mL)	Minimum Working Level (μg/mL)	Refs
HPLC	Biological samples	0.025~2.5	0.02	[13]
LC-MS/MS	Rat plasma	0.002~0.75	0.002	[14]
MEKC	human plasma	0.002~0.06	0.0005	[17]
DLLME	urine	0.0087~5	0.0027	[11]
potentiometry	blood-serum	0.000118~0.013	1.18 × 10^−4^	[21]
Spectrofluorimerty	Tabellae urine	0.004~0.9	0.0012	[41]
Spectrofluorimerty	Tabellae	0.000188~0.375	6.5 × 10^−5^	This work ^1^

^1^ Calculated with the molecular weight of AMA·HCl.

**Table 2:** Detecting result of simulative samples.

Setting Concentration of AMA (μg/mL)	Δ Int	Calculate Value (μg/mL)	Recovery (%) ± S.D ^1^
0.0282 (150 nM)	−144.06	0.028	99.3 ± 0.34
0.00469 (25 nM)	−24.86	0.00468	99.8 ± 0.78
0.000939 (5 nM)	−5.26	0.00089	94.8 ± 1.09

^1^ Average of five determinations; all measurements were performed in 50 mM NaOAc buffer (pH = 4.70).

## Supplementary Materials

The following are available online, Figures S1–S3: NMR spectrum of fluorescence indicator (ABAM hydrochloride); Figure S4: Competitive binding ^1^H NMR spectrum; Table S1: Results of the determination of *K*_rel_; Figures S5 & S6: The fluorescence intensity changes of ABAM and CB[7]·ABAM with different pH; Figures S7 & S8: Fluorescence spectra recorded for CB[7]·ABAM with addition of AMA; Figure S9: Fluorescence spectra recorded for CB[7]·ABAM in the presence of interference sample; Figures S10 & S11: Fluorescence spectra recorded for CB[7]·ABAM for determining the concentration of AMA in samples.
