# Supplementary material for: A Highly Selective and Strong Anti-Interference Host-Guest Complex as Fluorescent Probe for Detection of Amantadine by Indicator Displacement Assay
Source: Molecules. 2018 Apr 18;23(4):947. doi: 10.3390/molecules23040947 (PMC6017886; doi:10.3390/molecules23040947)
Supplement: Supplementary file 1 [file molecules-23-00947-s001.pdf]

Supporting Information

A highly-selective and strong anti-interference host-guest complex as fluorescent probe for detection of amantadine by indicator displacement assay

Linzhao Zhu, Zhiyong Zhao, Xiongzhi Zhang, Haijun Zhang, Feng Liang and Simin Liu*

The State Key Laboratory of Refractories and Metallurgy, School of Chemistry and Chemical Engineering, Wuhan University of Science and Technology, Wuhan 430081 (China)

Table of Contents		Page
Table of Contents	....	S1
^{1}H NMR, ^{13}C NMR, COSY NMR spectrum of ABAM	....	S2
Competitive binding ^{1}H NMR for determining K_a of CB[7]·ABAM	....	S3
The fluorescence intensity changes of ABAM and CB[7] ·ABAM with different pH	....	S4
Fluorescence titration graphs	....	S5
Fluorescence spectra for checking anti-interference capacity	....	S6
Fluorescence spectra for determination of AMA in simulative sample	....	S6
Fluorescence spectra for determination of AMA in pharmaceutical formulations	....	S7

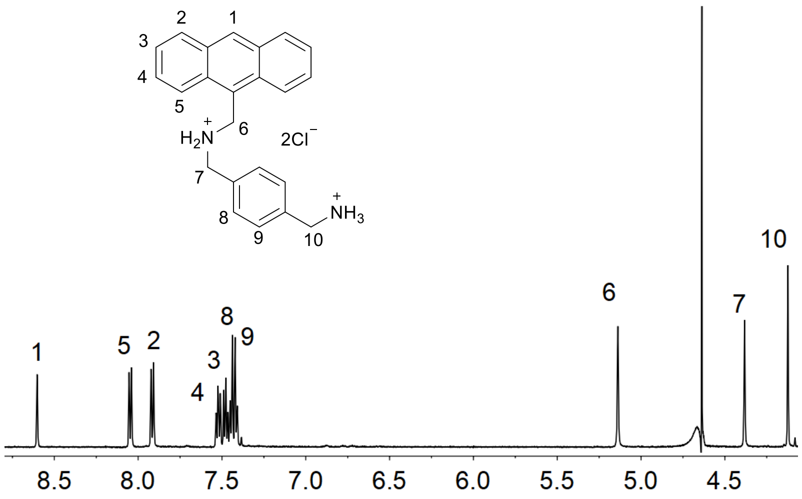

Figure S1 ^{1}H NMR spectrum (298 K, D_2O) of fluorescence indicator (ABAM hydrochloride).

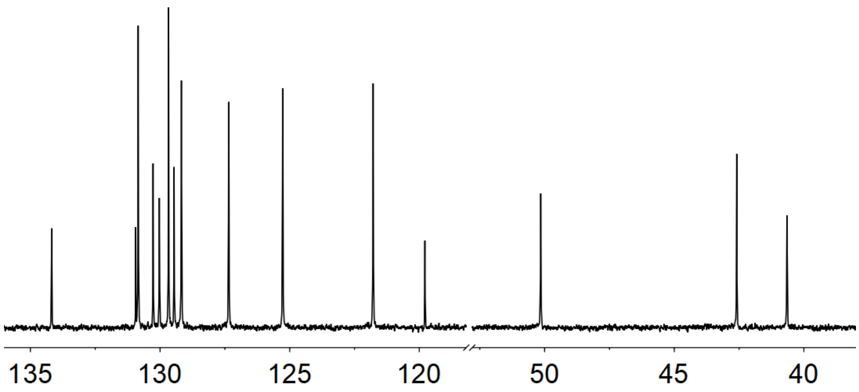

Figure S2 ^{13}C NMR spectrum (298 K, D_2O) of fluorescence indicator (ABAM hydrochloride)

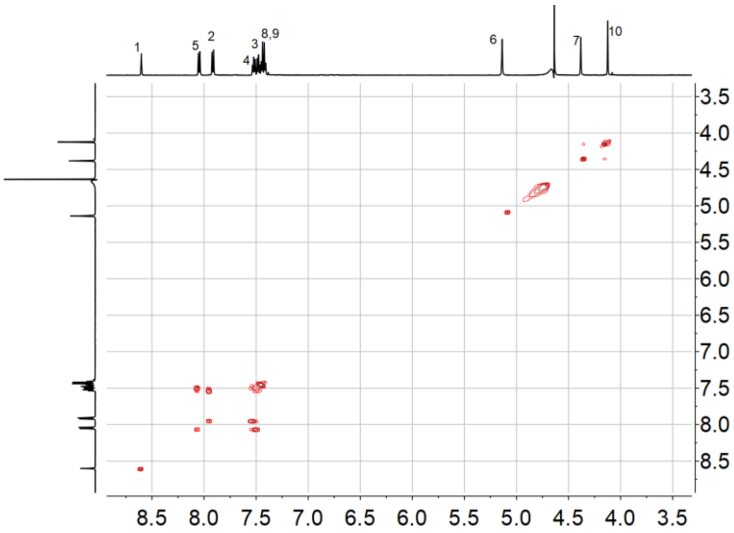

Figure S3 COSY NMR spectrum (298 K, D_2O) of ABAM.

Determination of K_{rel} for the Competition between ABAM and PXDA for CB[7].

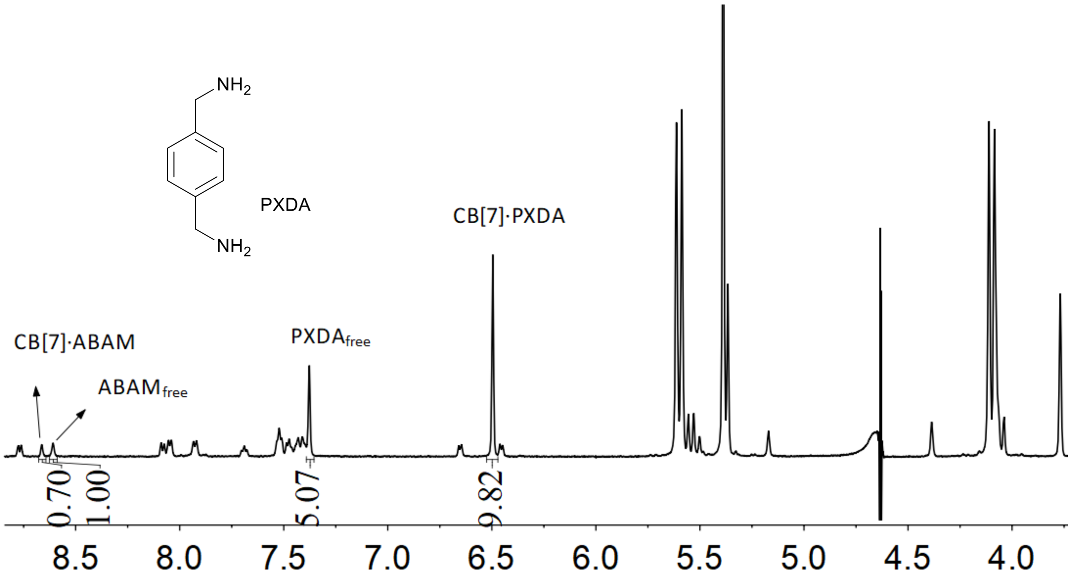

Figure S4 Competitive binding ^{1}H NMR spectrum used for the determination of K_{rel} between CB[7]·ABAM and CB[7]·PXDA (*p*-xylylenediamine) ([CB[7]] = [ABAM] = [PXDA] = 1.0 mM).

Table S1. Results of the determined K_{rel}

[CB[7]] : [ABAM] : [PXDA] (mM)	K_{rel}
1：1：1	2.07
1：0.8：0.8	2.15
1：1.2：1.2	2.10

The average K_{rel} is 2.11. The association constant of PXDA and CB[7] is 1.84 × 10^9 M^{-1} (*J. Am. Chem. Soc.*, **2005**, *127*, 15959−15967), the association constant of ABAM and CB[7] is 8.7 × 10^8 M^{-1}.

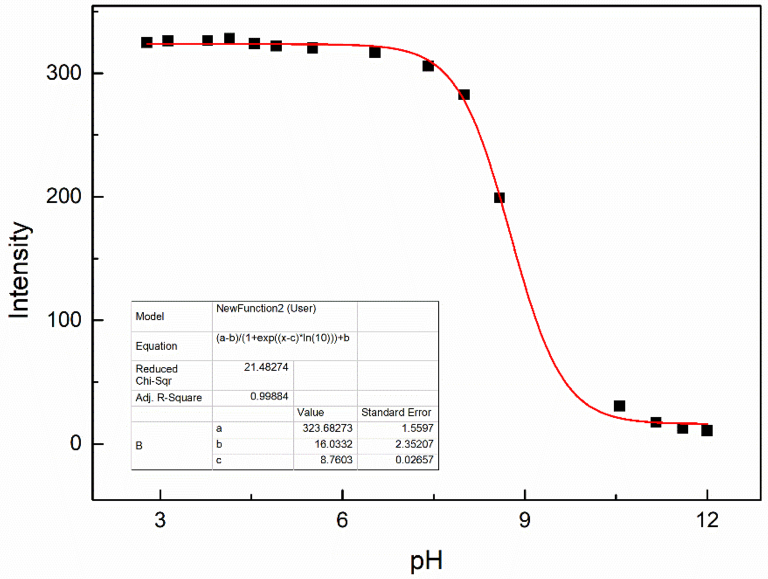

Figure S5 pH titration plots of the emission of ABAM (2.0 μM). The excitation and emission monochromator bandpasses were set at 15 nm and 3 nm, respectively ($\lambda_{ex}/\lambda_{em}$ = 366/417 nm). The fitting gives the acidity of ABAM as 8.8.

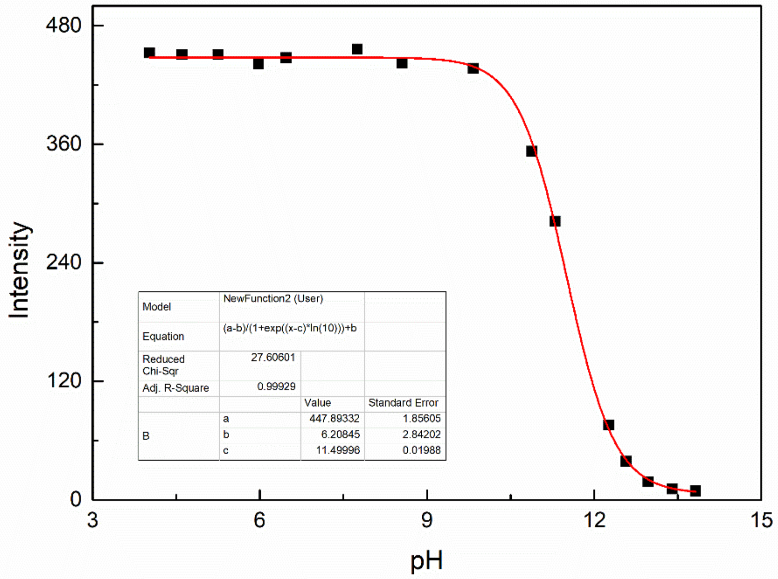

Figure S6 pH titration plots of the emission of ABAM in the presence of CB[7] ([ABAM] = [CB[7]] = 2.0 μM). The excitation and emission monochromator bandpasses were set at 15 nm and 3 nm, respectively ($\lambda_{ex}/\lambda_{em}$ = 366/417 nm). The fitting gives the acidity of CB[7]·ABAM as 11.5.

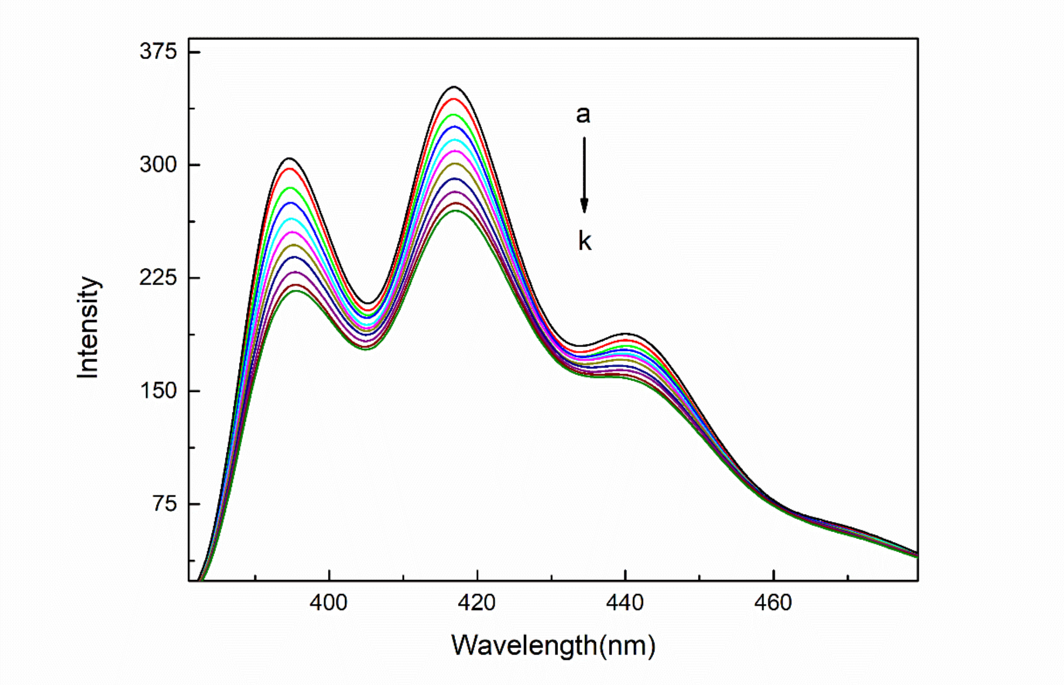

Figure S7 Fluorescence spectra recorded for CB[7]·ABAM (2.0 μM, pH = 4.70) with addition of AMA. The equivalent of AMA is: (a) 0; (b) 0.1; (c) 0.2; (d) 0.3; (e) 0.4; (f) 0.5; (g) 0.6; (h) 0.7; (i) 0.8; (j) 0.9; (k) 1.0. The excitation and emission monochromator bandpasses were set at 15 nm and 3 nm, respectively ($\lambda_{ex}/\lambda_{em}$ = 366/417 nm).

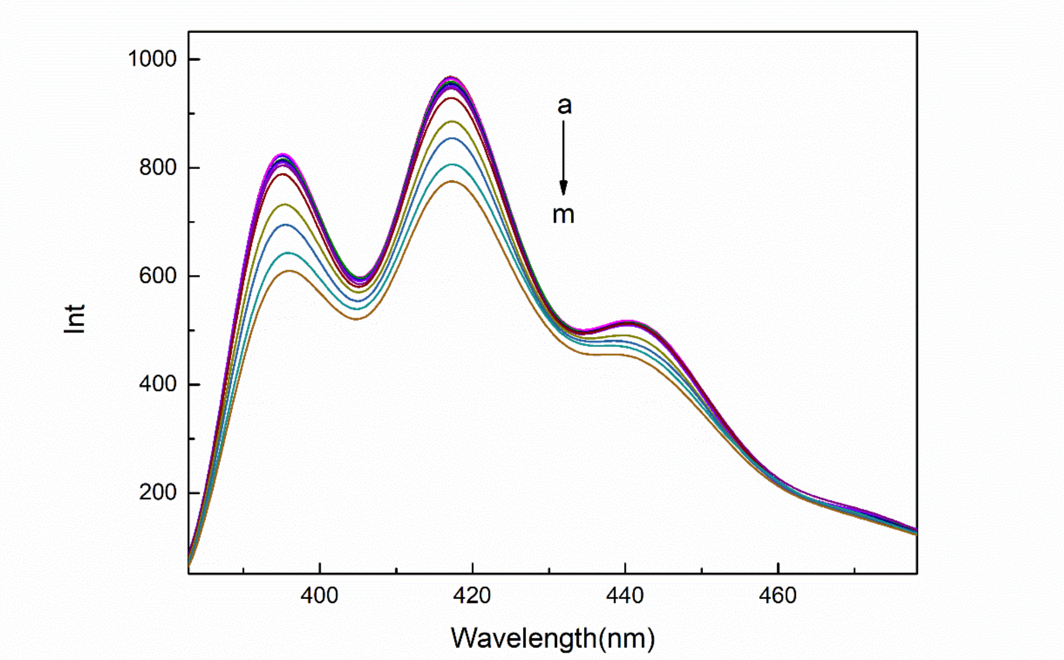

Figure S8 Fluorescence spectra recorded for CB[7]·ABAM (0.2 μM, pH = 4.70) with addition of AMA. The equivalent of AMA is (a): 0; (b) 0.005; (c) 0.01; (d) 0.02; (e) 0.04; (f) 0.06; (g) 0.08; (h) 0.1; (i) 0.2; (j) 0.4; (k) 0.6; (l) 0.8; (m) 1.0. The excitation and emission monochromator bandpasses were set at 10 nm and 6 nm, respectively ($\lambda_{ex}/\lambda_{em}$ = 366/417 nm).

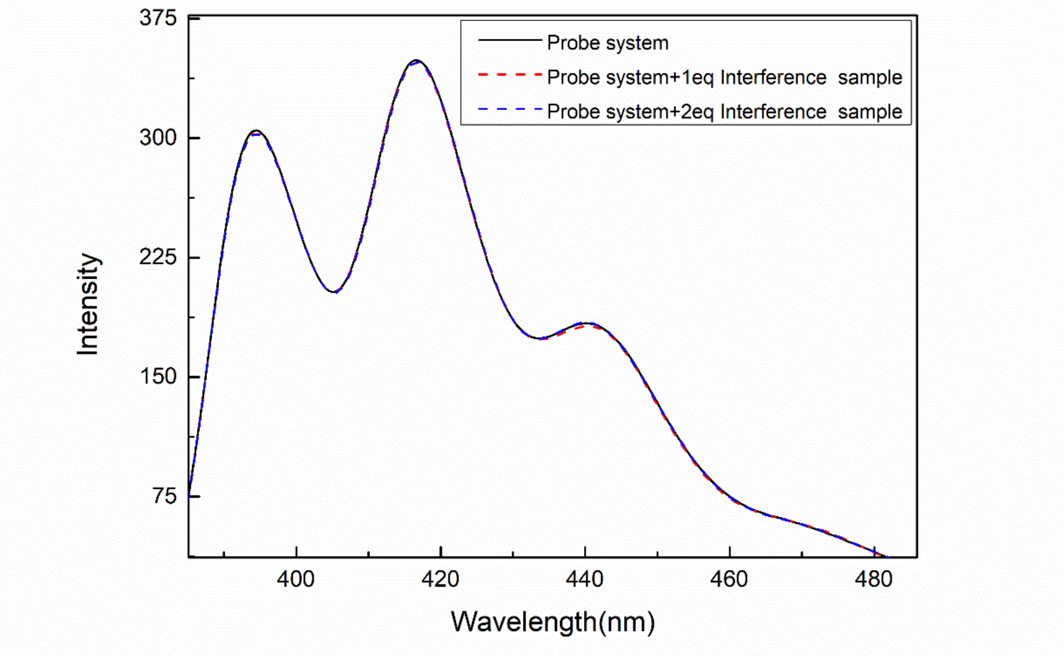

Figure S9 Fluorescence spectra recorded for CB[7]·ABAM (2.0 μM, pH = 4.70) in the presence of interference sample (interference sample is the mixture solution of ribavirin, doxycycline, levofloxacin and florfenicol; the concentration of each component in the mixture is the same). The excitation and emission monochromator bandpasses were set at 15 nm and 3 nm, respectively ($\lambda_{ex}/\lambda_{em}$ = 366/417 nm).

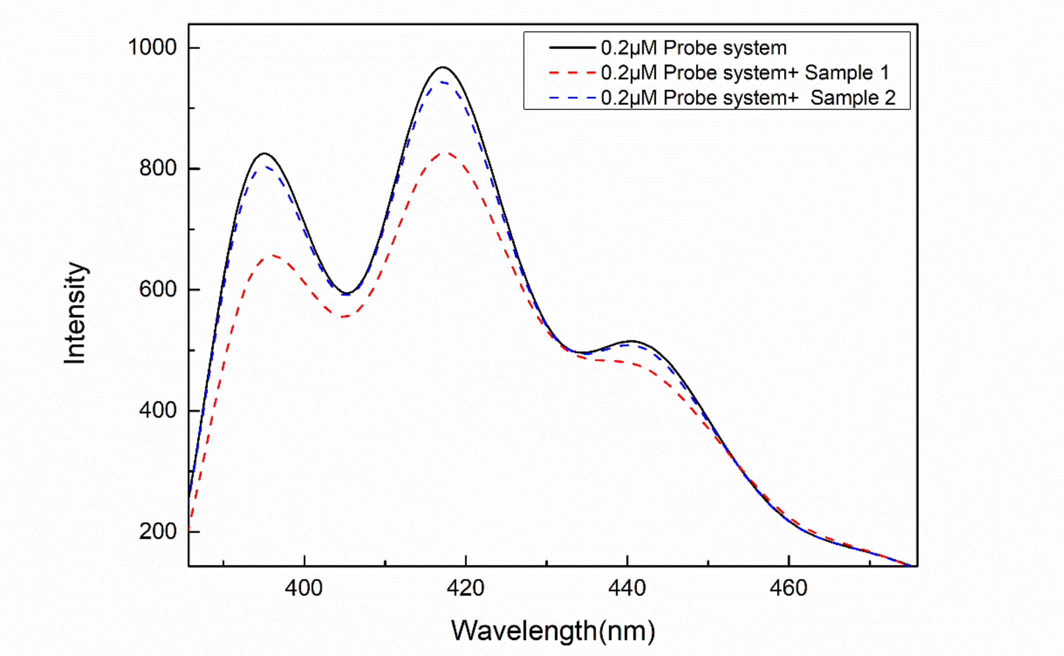

Figure S10 Fluorescence spectra recorded for CB[7]·ABAM (0.2 μM, pH = 4.70) in the presence of 150 nM AMA in simulative sample (red dash line) and 25 nM AMA in simulative sample (blue dash line). The excitation and emission monochromator bandpasses were set at 10 nm and 6 nm, respectively ($\lambda_{ex}/\lambda_{em}$ = 366/417 nm).

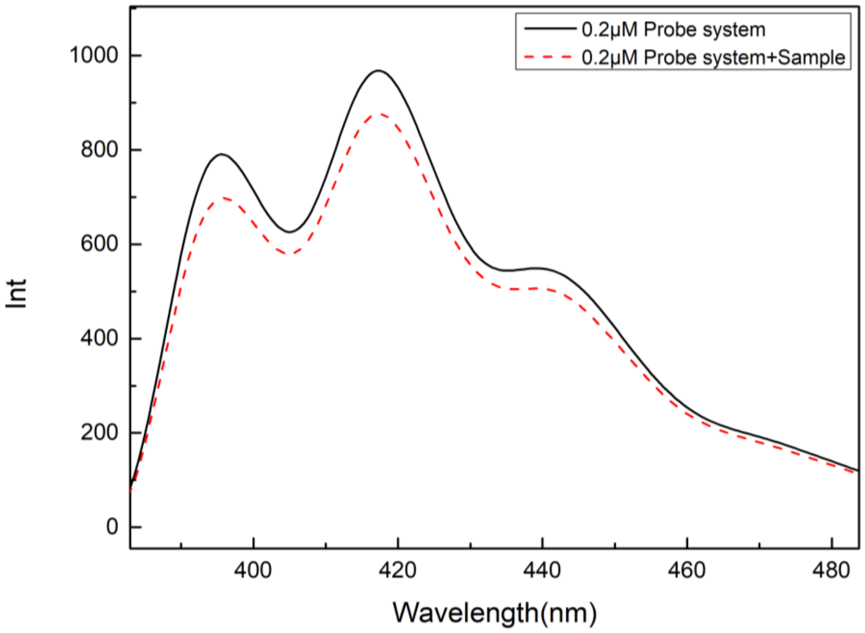

Figure S11 Fluorescence spectra recorded for CB[7]·ABAM (0.2 μM, pH = 4.70) in the presence of 100 nM AMA in pharmaceutical formulations sample. The excitation and emission monochromator bandpasses were set at 10 nm and 6 nm, respectively ($\lambda_{ex}/\lambda_{em}$ = 366/417 nm).
